# Stereotypic neutralizing V_H_ antibodies against SARS-CoV-2 spike protein receptor binding domain in patients with COVID-19 and healthy individuals

**DOI:** 10.1126/scitranslmed.abd6990

**Published:** 2021-01-04

**Authors:** Sang Il Kim, Jinsung Noh, Sujeong Kim, Younggeun Choi, Duck Kyun Yoo, Yonghee Lee, Hyunho Lee, Jongtak Jung, Chang Kyung Kang, Kyoung-Ho Song, Pyoeng Gyun Choe, Hong Bin Kim, Eu Suk Kim, Nam-Joong Kim, Moon-Woo Seong, Wan Beom Park, Myoung-don Oh, Sunghoon Kwon, Junho Chung

**Affiliations:** 1 Department of Biochemistry and Molecular Biology, Seoul National University College of Medicine, Seoul 03080, Republic of Korea.; 2 Ischemic/Hypoxic Disease Institute, Seoul National University Medical Research Center, Seoul 03080, Republic of Korea.; 3 Department of Electrical and Computer Engineering, Seoul National University, Seoul 08826, Republic of Korea.; 4 Department of Biomedical Science, Seoul National University College of Medicine, Seoul 03080, Republic of Korea.; 5 Department of Internal Medicine, Seoul National University College of Medicine, Seoul 03080, Republic of Korea.; 6 Department of Laboratory Medicine, Seoul National University College of Medicine, Seoul 03080, Republic of Korea.; 7 Interdisciplinary Program in Bioengineering, Seoul National University, Seoul 08826, Republic of Korea.; 8 BK21+ Creative Research Engineer Development for IT, Seoul National University, Seoul 08826, Republic of Korea.; 9 Biomedical Research Institute, Seoul National University Hospital, Seoul 03080, Republic of Korea.; 10 Institutes of Entrepreneurial BioConvergence, Seoul National University, Seoul 08826, Republic of Korea.; 11 Cancer Research Institute, Seoul National University College of Medicine, Seoul 03080, Republic of Korea.

## Abstract

Stereotypic antibody clonotypes exist in healthy individuals and may provide protective immunity against viral infections by neutralization. We observed that 13 of 17 patients with COVID-19 had stereotypic variable heavy chain (V_H_) antibody clonotypes directed against the receptor binding domain (RBD) of SARS-CoV-2 spike protein. These antibody clonotypes were composed of immunoglobulin heavy variable 3-53 (*IGHV3-53*) or *IGHV3-66* and immunoglobulin heavy joining 6 (*IGHJ6*) genes. These clonotypes included IgM, IgG3, IgG1, IgA1, IgG2, and IgA2 subtypes and had minimal somatic mutations, which suggested swift class switching after SARS-CoV-2 infection. The different IGHV chains were paired with diverse light chains resulting in binding to the RBD of SARS-CoV-2 spike protein. Human antibodies specific for the RBD can neutralize SARS-CoV-2 by inhibiting entry into host cells. We observed that one of these stereotypic neutralizing antibodies could inhibit viral replication in vitro using a clinical isolate of SARS-CoV-2. We also found that these V_H_ clonotypes existed in 6 of 10 healthy individuals, with IgM isotypes predominating. These findings suggest that stereotypic clonotypes can develop de novo from naïve B cells and not from memory B cells established from prior exposure to similar viruses. The expeditious and stereotypic expansion of these clonotypes may have occurred in patients infected with SARS-CoV-2 because they were already present.

## Neutralizing antibodies on hand

Stereotypic antibodies (Abs) are produced in healthy individuals by preexisting naïve B cells that have not undergone somatic hypermutation or class switching. Kim *et al.* have identified stereotypic neutralizing Abs (nAbs) against SARS-CoV-2 spike protein receptor binding domain (RBD) in healthy individuals and patients with COVID-19. They detected RBD-specific stereotypic variable heavy chain (V_H_) Ab clonotypes composed of Ig heavy variable 3-53 (*IGHV3-53*) or *IGHV3-66* and Ig heavy joining 6 (*IGHJ6*) genes in 13 of 17 patients with COVID-19. One stereotypic nAb could inhibit in vitro replication of a clinical isolate of SARS-CoV-2. These V_H_ clonotypes were also found in 6 of 10 healthy individuals with no evidence of exposure to SARS-CoV-2, and together, these findings provide evidence of the presence of preexisting nAbs to SARS-CoV-2.

## Teaser

Stereotypic neutralizing antibody clonotypes specific for SARS-CoV-2 receptor binding domain in patients with COVID-19 and healthy individuals.

## INTRODUCTION

Stereotypic antibodies (Abs), which share similar sequences across multiple individuals, can be produced in response to immunological stimulation upon infection. A subset of stereotypic neutralizing Abs (nAbs) encoded by naïve B cells that have not undergone somatic hypermutation and class switching from immunoglobulin M (IgM) or IgD isotypes are of great interest (*1*, *2*) because their origin effectively excludes the possibility that these nAbs evolved from preexisting clonotypes that are reactive to similar viruses. This phenomenon is referred to as original antigenic sin and has been linked to Ab-dependent enhancement (ADE) of viral infections, which can be potentially fatal, as observed during the development of experimental dengue virus vaccines (*3*–*6*). Several groups have identified nAbs for severe acute respiratory syndrome coronavirus 2 (SARS-CoV-2) (*7*–*11*), and one report suggests that stereotypic nAbs using germline Ig heavy variable 3-53 (*IGHV3-53*) and *IGHV3-66* genes may exist among convalescent patients with coronavirus disease 2019 (COVID-19) (*7*). The structural basis of the stereotypic nAb reaction to SARS-CoV-2 has been clarified with the cocrystal structure of two *IGHV3-53* nAbs in complex with the SARS-CoV-2 receptor binding domain (RBD), which defines the critical germline-encoded residues in the binding site of angiotensin-converting enzyme II (ACE2), the functional receptor of SARS-CoV-2 (*12*). The prevalence of these stereotypic nAb clonotypes among patients with SARS-CoV-2 and their characteristics, such as frequency within Ig repertoires, somatic mutations, isotypes, and chronological changes remains to be elucidated.

Here, we report stereotypic nAb clonotypes for SARS-CoV-2, which were identified by mapping nAbs onto deep Ig repertoires that were profiled from infected patients. The stereotypic naïve nAb clonotypes encoded by the *IGHV3-53*/*IGHV3-66* and Ig heavy joining 6 (*IGHJ6*) genes existed in the majority of convalescent patients with COVID-19 and showed little evidence of somatic mutations along with swift isotype class switching to IgG1, IgA1, and even IgA2 subtypes. Subsequently, we found that these same variable heavy chain (V_H_) clonotypes preexist predominantly as an IgM isotype in healthy individuals, possibly encoded by naïve B cells, which suggests that these stereotypic nAbs are not originated from clonotypes developed by previous infection with similar viruses. Individuals with these V_H_ clonotypes are may be able to rapidly produce potent nAbs and potentially afford partial protection against SARS-CoV-2.

## RESULTS

### Isolation and characterization of human nAbs

**Fig. 1:**
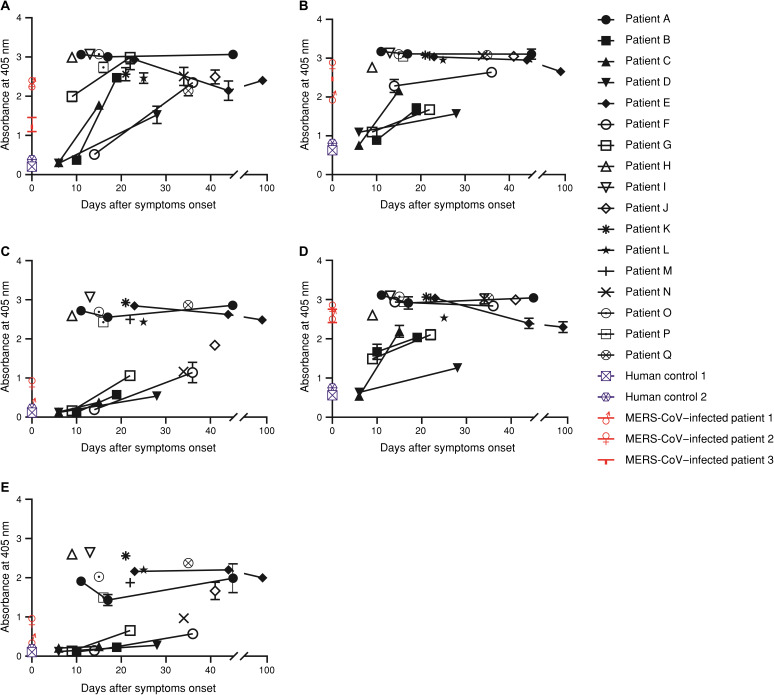
Titrations of serum IgG by ELISAs specific to SARS-CoV-2. Plasma samples from 17 patients with SARS-CoV-2 were diluted (1:100) and added to plates coated with recombinant SARS-CoV-2 (**A**) N, (**B**) S, (**C**) S1, (**D**) S2, which was fused to a polyhistidine (HIS) tag, or (**E**) RBD protein, which was fused to a human hCκ domain. The amount of bound IgG was determined using anti-human IgG (Fc-specific) antibody, and ABTS (2,2’-Azinobis [3-ethylbenzothiazoline-6-sulfonic acid]-diammonium salt) was used as the substrate. All experiments were performed in duplicate, and the data are presented as the means ± SD.

**Fig. 2:**
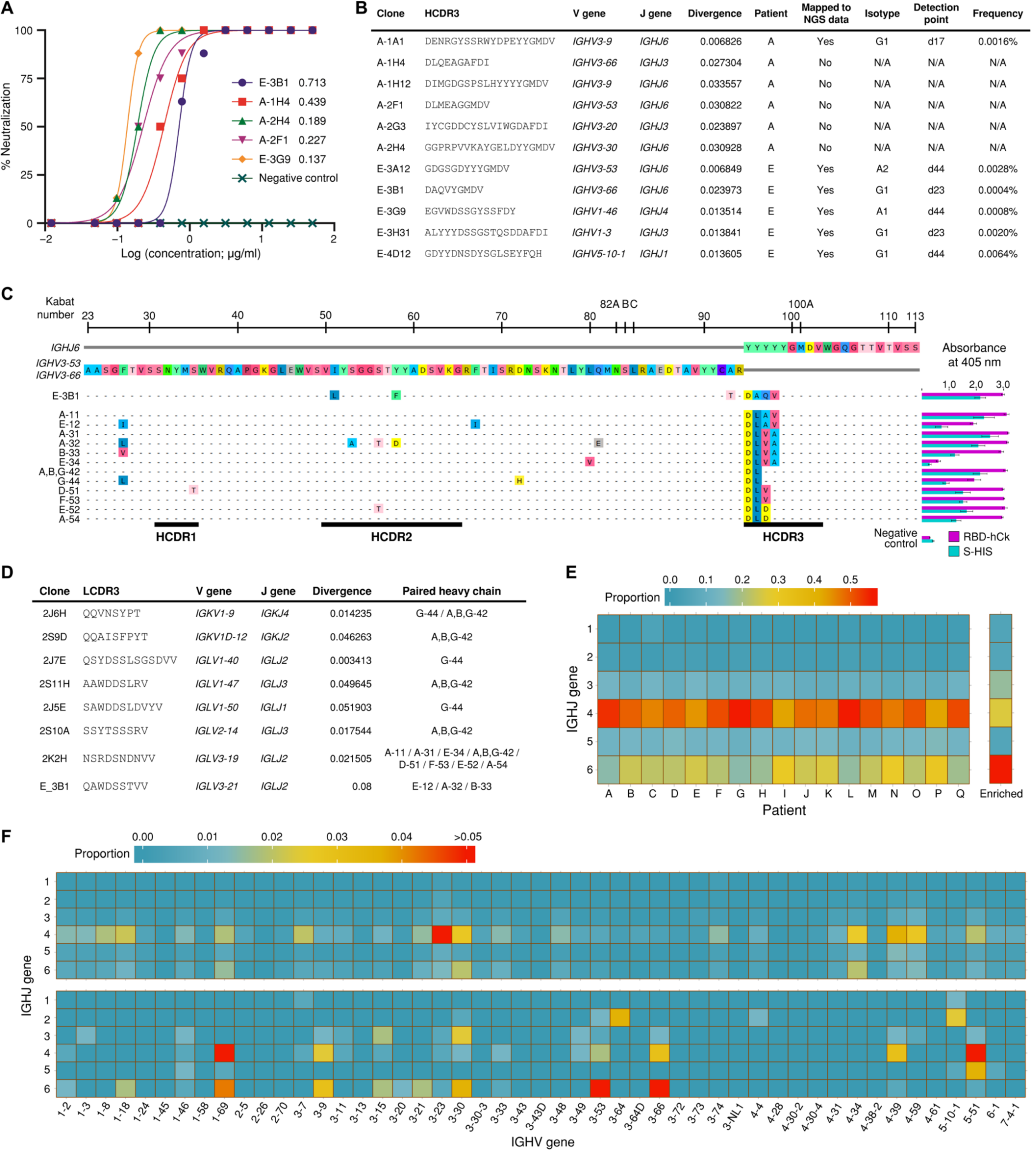
Characteristics of the isolated nAbs, stereotypic IGH clonotypes, and RBD binding–predicted clones. (**A**) Serially diluted IgG2/4 was mixed with an equal volume of SARS-CoV-2 containing 100 TCID_50_, and the IgG2/4-virus mixture was added to Vero cells with eight repeats and incubated for 5 days. Cells infected with 100 TCID_50_ of SARS-CoV-2, isotype IgG2/4 control, or without the virus were applied as positive, negative, and uninfected controls, respectively. CPE in each well was observed 5 days after infection. (**B**) Characteristics of nAbs found in patients A and E. (**C**) IGH clonotypes that are highly homologous to E-3B1 and reactive against recombinant SARS-CoV-2 S and RBD proteins. The right column shows the results of the phage ELISA. All experiments were performed in quadruplicate, and the data are presented as the means ± SD. (**D**) List of diverse Ig light chain clonotypes that can be paired with the IGH clonotypes from (B) to achieve reactivity. (**E**) J and (**F**) VJ gene usage in the IGH repertoire of patients (top) and the binding-predicted IGH clones (bottom). For the VJ gene usage heatmap, the frequency values for the IGH repertoire of all 17 patients were averaged and are displayed (top) along with those of the predicted RBD-binding IGH clones (bottom). N/A, not applicable.

To obtain monoclonal nAbs against SARS-CoV-2, we collected blood samples from 17 SARS-CoV-2–infected patients (patients A to Q), which were used to generate human Ab libraries. SARS-CoV-2 also uses spike (S) protein for receptor binding and membrane fusion in a mechanism similar to SARS-CoV (*13*) and requires binding with cellular receptor ACE2 to gain entry into the host cell (*14*, *15*). A previous report suggests that a human monoclonal Ab (mAb) that reacts with the RBD within the S1 region of the S protein can hinder the initial binding interaction between the virus and the cell, effectively neutralizing SARS-CoV-2 (*11*). We confirmed the reactivity of convalescent patient sera with recombinant SARS-CoV-2 S and RBD proteins. Patients A and E, who presented with extensive pneumonic infiltrates, also showed high plasma IgG titers against recombinant SARS-CoV-2 nucleocapsid (N), S, S1, S2, and RBD proteins, which was detected 11, 17, and 45 days after symptom onset in patient A and 23, 44, and 99 days after symptom onset in patient E (Fig. 1 and table S1). Sera samples from patients with Middle East respiratory syndrome coronavirus (MERS-CoV) cross-reacted with the SARS-CoV-2 S protein showed a higher titer against the S2 domain, and cross-reactivity to MERS-CoV S2 was detected in SARS-CoV-2 sera samples (Fig. 1 and fig. S1), suggesting the potential risk for ADE. We generated four human Ab libraries using a phage display system based on the blood samples from patient A, which were collected on days 17 and 45 (A_d17 and A_d45), and patient E, which were collected on days 23 and 44 (E_d23 and E_d44). We isolated 38 single-chain variable fragment (scFv) clones that were reactive against recombinant SARS-CoV-2 RBD as measured with SARS-CoV-2 RBD–specific enzyme-linked immunosorbent assay (ELISA) (fig. S2 and table S2). The half-maximal binding of scFv–human k light chain fragment (hCκ) to SARS-CoV-2 RBD occurred at concentrations ranging from 0.32 to 364 nM, which was comparable to findings in previous reports that have described human mAbs against the same protein (*8*, *11*). We tested whether these Ab clones could inhibit the binding between recombinant SARS-CoV-2 S protein and Vero E6 cells expressing the ACE2 receptor. A recombinant polyhistidine (HIS)–tagged SARS-CoV-2 S protein showed binding saturation at 200 nM when incubated with 1.5 × 10^5^ Vero E6 cells, as measured using flow cytometry analysis with a fluorescein isothiocyanate (FITC)–labeled anti-HIS Ab. For the analysis of different scFv clones, recombinant S protein (200 nM) was mixed with scFv–hIgG1 Fc region (hFc) fusion proteins, at a final concentration of either 200 nM (equimolar) or 600 nM (molar ratio of 1:3). Eleven clones (A-1A1, A-1H4, A-1H12, A-2F1, A-2H4, A-2G3, E-3A12, E-3B1, E-3G9, E-3H31, and E-4D12) almost completely inhibited binding between recombinant S protein and Vero E6 cells at 600 nM, and some showed inhibitory activity at 200 nM (fig. S3). We used an in vitro viral neutralization assay in which Vero cells were infected with a clinical isolate of SARS-CoV-2 (BetaCoV/Korea/SNU01/2020) encoding D614 in the viral S protein at a medium tissue culture infectious dose (TCID_50_) of 2500 and in the presence of 1 of the 11 scFv-hCκ fusion proteins, at concentrations of 0.5, 5, or 50 μg/ml. Viral RNA concentrations in the culture supernatant were determined 0, 24, 48, and 72 hours after infection. Nine scFv-hCκ fusion proteins exhibited complete neutralizing activity at 50 μg/ml (fig. S4), and two Abs (A-1H4 and E-3G9) showed potent neutralization even at 5 μg/ml (fig. S4). Five nAbs with IgG2/4 isotypes (E-3B1, A-1H4, A-2H4, A-2F1, and E-3G9) exhibited potent neutralizing activity against authentic SARS-CoV-2, with half-maximal inhibitory concentration (IC_50_) ranging from 0.137 to 0.713 μg/ml (Fig. 2A).

### Identification of stereotypic clonotypes from Ig heavy chain repertoire of SARS-CoV-2–infected patients

We performed deep profiling of the Ig repertoire in three chronological blood samples each from patients A and E, two chronological samples each from patients B, C, D, F, and G, and a single time point sample from the remaining 10 patients (H to Q). We searched for nAb clonotypes that had identical variable (V) and joining (J) gene combinations and perfectly matched heavy chain complementarity–determining region 3 (HCDR3) amino acid sequences among the Ig heavy chain (IGH) repertoires of patients A and E. One and five nAb clonotypes that met these criteria were identified in patients A and E, respectively (Fig. 2B), and three nAbs (A-2F1, E-3A12, and E-3B1) were encoded by *IGHV3-53/IGHV3-66* and *IGHJ6* (Fig. 2B). These two V_H_ genes, IGHV3-53*01 and IGHV3-66*01, share an identical amino acid sequence, except for the H12 residue (isoleucine in *IGHV3-53* and valine in *IGHV3-66*), and only five nucleotides differ between their sequences. Four clonotypes were IgG1, and two clonotypes were class-switched to IgA1 and IgA2, when examined 44 days after symptom onset (Fig. 2B). These clonotypes had a very low frequency of somatic mutations (1.03 ± 0.51%), which was compatible with findings about other nAbs in previous reports (*7*, *8*). We evaluated all V_H_ sequences from the 17 patients and searched the clonotypes of 11 nAbs that were encoded by the same VJ genes and showed 66.6% or higher identity in the amino acid sequence for HCDR3 (fig. S5). Clonotypes that were highly homologous to the E-3B1 nAb were found among 13 of 17 patients, with a total of 126 clonotypes having the isotype of IgG3 (patients I, K, and P), IgG1 (patients A, B, D to I, K, M, O, and P), IgA1 (patients E, G, I, and J), IgG2 (patients I to K), and IgA2 (patient E) (table S3). These clonotypes shared nearly identical V_H_ sequences (92.45 ± 3.04% identity between amino acid sequences), with E-3B1 displaying an extremely low frequency of somatic mutations (0.98 ± 1.48%). Among these 126 clonotypes, 43 unique HCDR3s were identified by amino acid sequence, and 12 unique HCDR3s were present in more than one patient (Table 1).

**Table 1:** The stereotypic V_H_ clonotypes against SARS-CoV-2 RBD in the healthy population and in SARS-CoV-2–infected patients. The healthy population samples based on publicly available IGH repertoires or patient identification can be found in the sample column. Clonotypes were mapped according to identical VJ gene usage of *IGHV3-53/IGHV3-66* and *IGHJ6* and perfectly matched HCDR3 amino acid sequences. Read counts of the mapped sequences in the repertoires of each sample were annotated in the occurrence column. For clonotypes with multiple occurrences, the means and SD of divergence were represented. The proportion of each isotype is indicated for each sample as a percentage.

**Healthy population**						
**Sample**	**V gene**	**J gene**	**CDR3 amino acid**	**Divergence**	**Isotype**	**Occurrence**
326650	*IGHV3-53/3-66*	*IGHJ6*	DLYYYGMDV	0.007 ± 0.003	M (100%)	12
326713	*IGHV3-53/3-66*	*IGHJ6*	DLYYYGMDV	0.005 ± 0.010	M (92.3%), G (7.7%)	13
326780	*IGHV3-53/3-66*	*IGHJ6*	DLYYYGMDV	0.014 ± 0.010	M (97.4%), G (2.6%)	38
326797	*IGHV3-53*	*IGHJ6*	DLYYYGMDV	0.004	M (100%)	1
327059	*IGHV3-53/3-66*	*IGHJ6*	DLYYYGMDV	0.003 ± 0.005	M (100%)	8
D103	*IGHV3-53*	*IGHJ6*	DLYYYGMDV	0.008 ± 0.020	M (100%)	9
326650	*IGHV3-53/3-66*	*IGHJ6*	DLDYYGMDV	0.006 ± 0.002	M (75%), G (25%)	4
326713	*IGHV3-53/3-66*	*IGHJ6*	DLDYYGMDV	0.012 ± 0.018	M (100%)	4
326797	*IGHV3-66*	*IGHJ6*	DLDYYGMDV	0.055	M (100%)	1
327059	*IGHV3-53/3-66*	*IGHJ6*	DLDYYGMDV	0.001 ± 0.002	M (100%)	4
D103	*IGHV3-53*	*IGHJ6*	DLDYYGMDV	0.053	M (100%)	1
326713	*IGHV3-53/3-66*	*IGHJ6*	DLVAYGMDV	0.008 ± 0.011	M (100%)	2
326713	*IGHV3-53*	*IGHJ6*	DLVYYGDMV	0.001 ± 0.002	M (100%)	3
326797	*IGHV3-53*	*IGHJ6*	DLVYYGMDV	0.089 ± 0.008	M (100%)	2
326713	*IGHV3-53*	*IGHJ6*	DLVVYGMDV	0.024 ± 0.052	M (100%)	5
326780	*IGHV3-53/3-66*	*IGHJ6*	DLSYYGMDV	0.024 ± 0.024	M (98.44%), D (0.78%), G (0.78%)	128
D103	*IGHV3-53*	*IGHJ6*	DLSYYGMDV	0.022 ± 0.003	M (100%)	2
327059	*IGHV3-53*	*IGHJ6*	DLGDYGMDV	0.000	M (100%)	1
326713	*IGHV3-66*	*IGHJ6*	DAVSYGMDV	0.000 ± 0.000	M (100%)	2
**SARS-CoV-2–infected patients**						
**Sample**	**V gene**	**J gene**	**CDR3 amino acid**	**Divergence**	**Isotype**	**Occurrence**
A	*IGHV3-53*	*IGHJ6*	DLYYYGMDV	0.002 ± 0.004	M (5.1%), G1 (94.9%)	59
B	*IGHV3-53*	*IGHJ6*	DLYYYGMDV	0.000 ± 0.000	M (33.3%), G1 (66.7%)	3
G	*IGHV3-53/3-66*	*IGHJ6*	DLYYYGMDV	0.005 ± 0.003	G1 (84.6%), A1 (15.4%)	14
I	*IGHV3-53*	*IGHJ6*	DLYYYGMDV	0.000 ± 0.000	M (100%)	4
K	*IGHV3-53*	*IGHJ6*	DLYYYGMDV	0.009 ± 0.000	G1 (100%)	2
A	*IGHV3-53*	*IGHJ6*	DLAVYGMDV	0.004 ± 0.000	G1 (100%)	2
E	*IGHV3-66*	*IGHJ6*	DLAVYGMDV	0.018 ± 0.000	G1 (100%)	6
A	*IGHV3-53*	*IGHJ6*	DLDYYGMDV	0.000 ± 0.000	G1 (100%)	3
E	*IGHV3-53*	*IGHJ6*	DLDYYGMDV	0.004 ± 0.000	A1 (100%)	4
I	*IGHV3-66*	*IGHJ6*	DLDYYGMDV	0.002 ± 0.003	G1 (100%)	5
K	*IGHV3-53*	*IGHJ6*	DLDYYGMDV	0.007 ± 0.005	G1 (100%)	107
M	*IGHV3-53*	*IGHJ6*	DLDYYGMDV	0.018	G1 (100%)	1
A	*IGHV3-53*	*IGHJ6*	DLVAYGMDV	0.008 ± 0.017	G1 (100%)	14
B	*IGHV3-53*	*IGHJ6*	DLVAYGMDV	0.009	G1 (100%)	1
E	*IGHV3-53*	*IGHJ6*	DLVAYGMDV	0.005 ± 0.002	G1 (100%)	6
D	*IGHV3-53*	*IGHJ6*	DLVYYGMDV	0.004	G1 (100%)	1
E	*IGHV3-53*	*IGHJ6*	DLVYYGMDV	0.013	A1 (100%)	1
F	*IGHV3-53*	*IGHJ6*	DLVYYGDMV	0.001 ± 0.003	M (75%), G1 (25%)	16
B	*IGHV3-53*	*IGHJ6*	DLVVYGMDV	0.002 ± 0.002	M (27.3%), G1 (72.7%)	11
E	*IGHV3-53*	*IGHJ6*	DLVVYGMDV	0.013 ± 0.000	A2 (100%)	4
H	*IGHV3-53*	*IGHJ6*	DLVVYGMDV	0.009 ± 0.000	G1 (100%)	7
A	*IGHV3-53*	*IGHJ6*	DLSYYGMDV	0.013 ± 0.016	G1 (100%)	5
F	*IGHV3-53*	*IGHJ6*	DLSYYGMDV	0.018	G1 (100%)	1
O	*IGHV3-53*	*IGHJ6*	DLSYYGMDV	0.000	G1 (100%)	1
A	*IGHV3-53*	*IGHJ6*	DLGDYGMDV	0.009 ± 0.000	G1 (100%)	3
E	*IGHV3-53*	*IGHJ6*	DLGDYGMDV	0.018 ± 0.019	G1 (85.7%), A1 (14.3%)	7
F	*IGHV3-53*	*IGHJ6*	DLGDYGMDV	0.003 ± 0.002	M (92.0%), G1 (8.0%)	163
H	*IGHV3-53*	*IGHJ6*	DLGDYGDMV	0.004 ± 0.000	G1 (100%)	8
G	*IGHV3-53*	*IGHJ6*	DAVSYGMDV	0.004 ± 0.004	M (7.0%), G1 (93.0%)	57
I	*IGHV3-53*	*IGHJ6*	DAVSYGMDV	0.007 ± 0.003	G1 (100%)	9
P	*IGHV3-53*	*IGHJ6*	DAVSYGMDV	0.000 ± 0.000	G1 (100%)	3
E	*IGHV3-53*	*IGHJ6*	DLGPYGMDV	0.009	G1 (100%)	1
I	*IGHV3-53/3-66*	*IGHJ6*	DLGPYGMDV	0.010 ± 0.003	G3 (40%), G1 (40%), A1 (20%)	4
A	*IGHV3-53*	*IGHJ6*	DLVIYGMDV	0.003 ± 0.004	M (5.9%), G1 (94.1%)	17
I	*IGHV3-66*	*IGHJ6*	DLVIYGMDV	0.007 ± 0.004	G1 (100%)	8
E	*IGHV3-53/3-66*	*IGHJ6*	DLVVLGMDV	0.009 ± 0.000	A2 (100%)	20
I	*IGHV3-53*	*IGHJ6*	DLVVLGMDV	0.000	G1 (100%)	1

### Light chain plasticity of the stereotypic V_H_ clonotypes for binding to SARS-CoV-2 RBD

To test the reactivity of clonotypes homologous to E-3B1 against the SARS-CoV-2 S protein, we arbitrarily sampled 12 IGH clonotypes (Fig. 2C), containing five different HCDR3s, from the IGH repertoires of 13 patients. The genes encoding these IGH clonotypes were chemically synthesized and used to construct scFv genes with the variable λ chain (V_λ_) gene from the E-3B1 clone. The reactivity of these scFv clones to recombinant S and RBD was tested using a phage ELISA, and three clones (E-12, A-32, and B-33) reacted against recombinant S and RBD proteins (Fig. 2C). scFv libraries were constructed, using the A-11, A-31, E-34, A,B,G-42, G-44, D-51, F-53, E-52, and A-54 genes, and the variable k chain (V_κ_)/V_λ_ genes amplified from patients A, E, and G, and all 12 IGH clonotypes were reactive against both recombinant S and RBD proteins when paired with eight different V_κ_ and V_λ_ genes (Fig. 2, C and D). All seven light chain–profiled patients (A to G) had these V_κ_/V_λ_ clonotypes with identical VJ gene usage and perfectly matching light chain CDR3 (LCDR3) amino acid sequences (fig. S6). Ig lambda variable 2-14 (*IGLV2-14*)/Ig lambda joining 3 (*IGLJ3*), *IGLV3-19*/*IGLJ2*, and *IGLV3-21*/*IGLJ2* were frequently used across all seven patients (figs. S7 and S8). Because E-3B1 effectively inhibited the replication of SARS-CoV-2 (Fig. 2A), these 126 clonotypes are likely to neutralize SARS-CoV-2 when paired with an optimal light chain.

### Stereotypic naïve IGH clonotype against SARS-CoV-2 preexist in the healthy population

Among IGH clonotypes, A,B,G-42 was unique, presenting little to no evidence of somatic mutations (0.6 ± 0.8%) and containing an HCDR3 (DLYYYGMDV) formed by the simple joining of *IGHV3-53* and *IGHJ6*. This naïve V_H_ sequence existed in the IGH repertoire of five patients (patients A, B, G, I, and K), as IgM and IgG1, IgM and IgG1, IgG1 and IgA1, IgM, or IgG1 subtypes, respectively (Table 1). The IGH clonotypes encoded by *IGHV3-53*/*IGHV3-66* and *IGHJ6* that had an HCDR3 (DLYYYGMDV) with zero to one somatic mutation could be identified within the IGH repertoire of 6 of 10 healthy individuals, predominantly as an IgM isotype (*16*), based on publicly available IGH repertoires (Table 1). The A,B,G-42 clonotype showed light chain plasticity and could pair with five V_κ_ /V_λ_ genes to achieve RBD binding. In particular, the V_κ_ gene (2J6H) accumulated only five somatic mutations (1.4% divergence). None of the 12 clones, including A,B,G-42, reacted against the recombinant RBD proteins from either SARS-CoV or MERS-CoV (fig. S9). None of the 37 identified MERS-RBD–binding human mAbs (from two patients) were encoded by *IGHV3-53/IGHV3-66* and *IGHJ6* (table S4) based on analysis of a prior study (*17*). Therefore, the presence of these stereotypic naïve IGH clonotypes in the healthy population, and their light chain plasticity needed to achieve SARS-CoV-2 RBD binding, may be unique to SARS-CoV-2, which might provide a rapid and effective humoral response to the virus among patients who express these clonotypes. These findings suggest that a portion of the population has germline precursor B cells, encoded by *IGHV3-53*/*IGHV3-66* and *IGHJ6*, which can actively initiate virus neutralization upon SARS-CoV-2 infection.

### Distinctive V and J gene usage of the SARS-CoV-2 RBD-binding Abs

To further elucidate the preferential use of *IGHV3-53/IGHV3-66* and *IGHJ6* genes during the generation of SARS-CoV-2 RBD-binding Abs, we extracted 252 predicted RBD-binding clones from our biopanning data. We previously showed that Ab clones with binding properties can be predicted using next-generation sequencing (NGS) technology and analyzing the enrichment patterns of biopanned clones (*18*, *19*). The *IGHJ4* gene was more prominent within the IGH repertoires of 17 patients, similar to healthy human samples (*16*, *20*), but the predicted RBD-binding clones primarily used the *IGHJ6* gene (Fig. 2E). Furthermore, the predicted RBD-binding clones showed the dominant usage of *IGHV3-53*/*IGHJ6* and *IGHV3-66*/*IGHJ6* pairs, which was not observed in the whole IGH repertoires of patients (Fig. 2F).

### Chronological follow-up of IGH repertoire and the SARS-CoV-2 RBD-binding Abs from patients

**Fig. 3:**
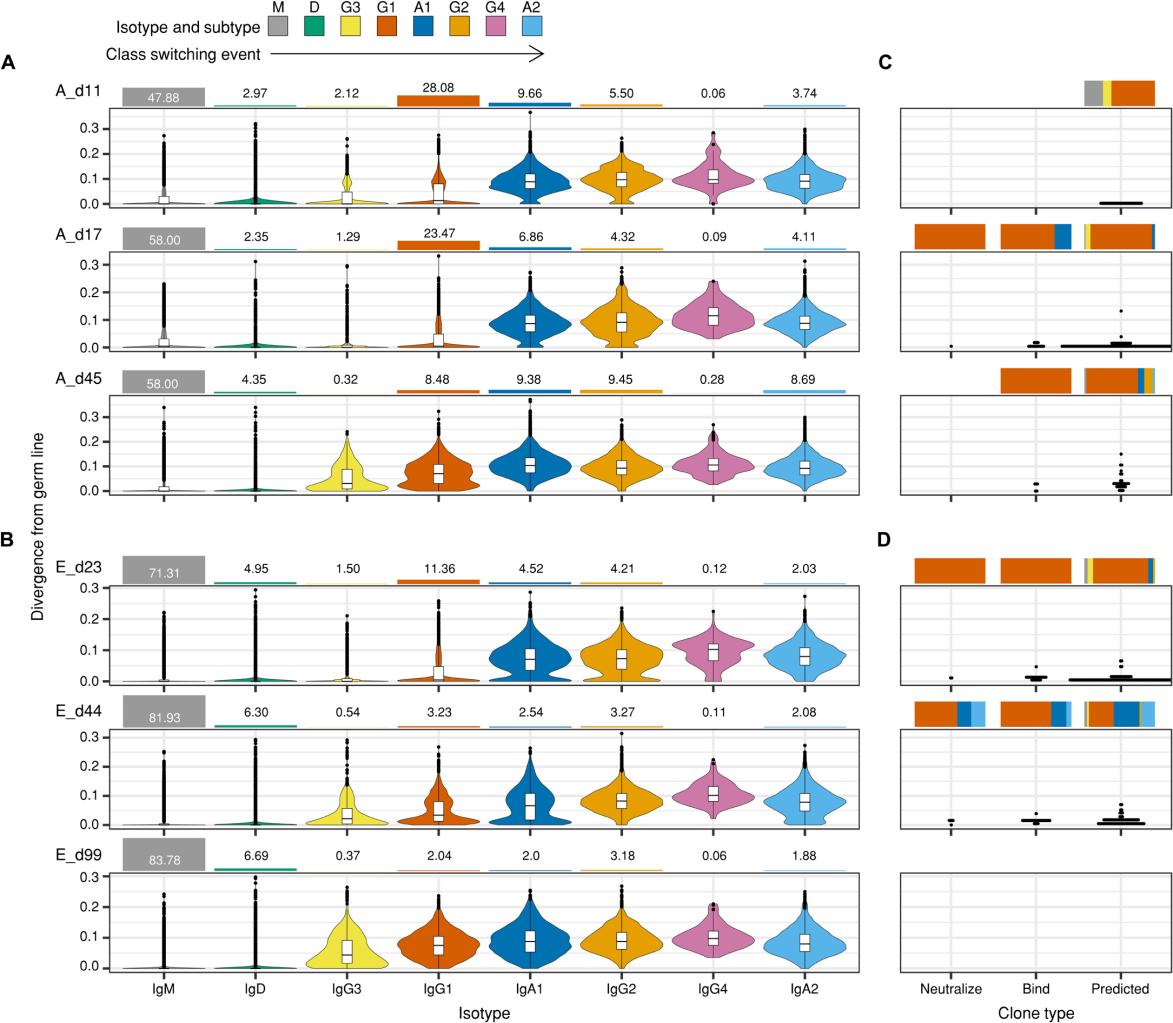
Deep profiling of the IGH repertoires of patients A and E. (**A** and **B**) IGH repertoires of (A) patient A and (B) patient E were analyzed 11, 17, and 45 (A_d11, A_d17, and A_d45) days and 23, 44, and 99 (E_d23, E_d44, and E_d99) days after symptom onset, respectively. IGH repertoires were examined according to divergence from the germ line and the isotype composition of the sequences. Values for divergence from the germ line were calculated separately for each isotype and are presented as violin plots, ordered by the class-switching event. The bar graphs on the top of the violin plots represent the proportion of each isotype in the repertoire. (**C** and **D**) Mapping of three types of RBD-binding IGH sequences (neutralize, bind, and predicted), derived from either (C) patient A or (D) patient E, against the corresponding IGH repertoire. The positions of the RBD-binding IGH sequences in the divergence value were annotated as dot plots on the same scale used for (A) and (B). Bar graphs on the top of the dot plots indicate the isotype compositions of the sequences in the repertoire.

Naïve B cells typically undergo somatic hypermutations, clonal selection, and class switching after antigen exposure. We examined the chronological events that occurred in all IGH clonotypes identified in patients A to G and those that were reactive against the SARS-CoV-2 RBD. In the entire patient IGH repertoire, naïve-derived IGH clonotypes with minimal somatic mutations (<2.695 ± 0.700%) showed increased IgG3 and IgG1 subtypes, and the proportion of the IgG1 subtype was markedly increased for a period (Fig. 3, A and B, and fig. S10). The naïve-derived IGH clonotypes were detected as minor populations of IgA1 and IgG2 subtypes in patients A and E (Fig. 3, A and B) and as an IgA2 subtype in patient E (Fig. 3B). We categorized RBD-reactive clones into three groups: (i) nAbs (neutralize), (ii) binding-confirmed Abs (bind), and (iii) binding-predicted Abs (predicted). In all three groups, these IGH clonotypes appeared and disappeared throughout the disease course, showed a low frequency of somatic mutations (Fig. 3, C and D), and displayed rapid class switching, especially to IgG1, IgA1, and IgA2. These results suggest that RBD-reactive IGH clonotypes can emerge rapidly and undergo class switching to IgG1, IgA1, and IgA2, without accumulating many somatic mutations. This marked temporal surge of naïve IGH clonotypes, with rapid class switching, occurred across the entire IGH repertoire of the patients and was not confined to those reactive to the SARS-CoV-2 RBD.

### Selected nAbs retained its ability to bind to most current SARS-CoV-2 mutants

**Fig. 4:**
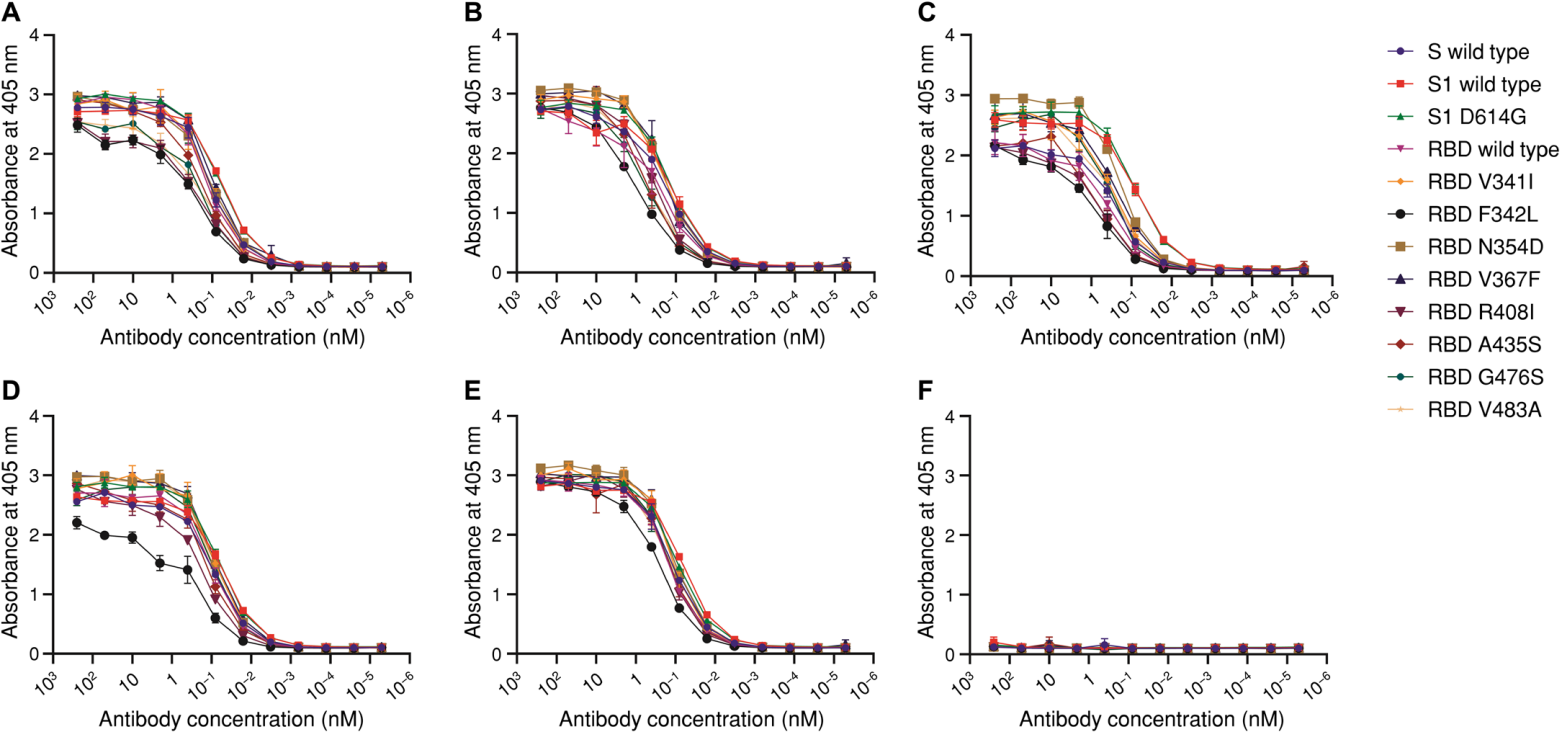
Reactivity of nAbs against recombinant SARS-CoV-2 spike mutants. Recombinant wild-type or mutant (V341I, F342L, N354D, V367F, R408I, A435S, G476S, V483A, and D614G) SARS-CoV-2 S, S1, or RBD protein–coated microtiter plates were incubated with varying concentrations of (**A**) E-3B1-hFc, (**B**) A-1H4-hFc, (**C**) A-2F1-hFc, (**D**) A-2H4-hFc, (**E**) E-3G9-hFc, and (**F**) irrelevant scFv-hFc. HRP-conjugated anti-human IgG antibody was used as the probe, and ABTS was used as the substrate. All experiments were performed in triplicate, and data are presented as the means ± SD.

Several mutations within the S1 domain of the SARS-CoV-2 S protein have been identified during the 2019–2020 global pandemic (*21*); thus, we examined the probability of escape mutants emerging from the IGH repertoire induced by the wild-type virus infection. Our E-3B1, A-1H4, A-2F1, A-2H4, and E-3G9 nAbs successfully bound to different recombinant mutant S1 proteins (V341I, F342L, N354D, V367F, R408I, A435S, G476S, V483A, and D614G) in a dose-dependent manner, with compatible reactivity against recombinant wild-type S1 and RBD proteins (Fig. 4). Therefore, the human IGH immune repertoire may provide effective protection against an array of SARS-CoV-2 mutants.

## DISCUSSION

In response to SARS-CoV-2 infection, some human IGH repertoires can efficiently generate clonotypes encoded by *IGHV3-53/IGHV3-66* and *IGHJ6*, which can pair with diverse light chains, and form Abs with both RBD binding and virus neutralization properties. These clonotypes appear to have few to no somatic mutations and can undergo swift class switching to IgG1, IgA1, and even IgA2 subtypes. The expeditious development of these IGH clonotypes is possible because the naïve stereotypic *IGHV3-53/IGHV3-66* and *IGHJ6* clonotypes preexist in a portion of the healthy population, predominantly as an IgM isotype. In line with our findings, several groups have reported potent human nAbs, composed of either *IGHV3-53* or *IGHV3-66* and *IGHJ6* genes, using single B cell sequencing technology (*7*–*11*). The crystal structures of two *IGHV3-53* nAbs has been determined, which has shown that two key motifs within HCDR1 and HCDR2 that are encoded in the *IGHV3-53* germ line can bind to SARS-CoV-2 S protein RBD (*12*). Therefore, the preferential use of *IGHV3-53/IGHV3-66* and *IGHJ6* in the development of nAbs to SARS-CoV-2 appears promising, especially given evidence that *IGHV3-53/IGHV3-66* and *IGHJ6* are able to pair with diverse light chains to form nAbs that can bind to the RBD. We found eight different light chains from our experiments, and other groups have identified nine different light chains (*12*). It is expected that the extent of light chain plasticity is broad enough for virus-exposed individuals to successfully evolve nAbs, given that class-switched *IGHV3-53/IGHV3-66* and *IGHJ6* clonotypes were present in 13 of 17 patients from our study.

Now, we do not know whether the stereotypic nAbs are polyreactive or autoreactive. Rather, our selected stereotypic nAbs, including A,B,G-42, do not appear to cross-react with recombinant RBD proteins of either SARS-CoV or MERS-CoV. Several reports have evaluated autoreactivity or polyreactivity of SARS-CoV-2 nAbs with minimal somatic mutations, and one report showed that 18 nAbs, including four Abs encoded by *IGHV3-53/IGHJ4*, *IGHV3-53/IGHJ6*, or *IGHV3-66/IGHJ4*, did not show evidence of being polyreactive or autoreactive (*22*). Another group characterized 29 Abs, including three nAbs encoded by *IGHV3-53*, with limited somatic mutations (<1.4%) and a HCDR3 length of 10 or 16 amino acids, and none of these Abs were polyreactive (*9*). In contrast, it was recently reported that one of three mAbs encoded by *IGHV3-53*, with limited somatic mutations (1.4%), reacted with the Ebola glycoprotein and HIV-1 gp140, and one of the four nAbs encoded by *IGHV3-66* with limited somatic mutations (0.3%) showed moderate autoreactivity (*23*). On the basis of these observations, it is likely that autoreactivity and polyreactivity may depend on specific somatic mutations and HCDR3 sequences.

We analyzed a possible correlation between clinical features and Ab responses of 17 individuals who were infected with SARS-CoV-2. Of 17 laboratory-confirmed patients, two patients (patients M and O) had a severe respiratory illness that required mechanical ventilation and six patients (patients A, H, I, K, L, and P) with moderate illness required supplemental oxygenation. These eight patients with relatively severe clinical courses had high titers of IgG Ab against SARS-CoV-2. However, three patients (patients E, J, and Q) with mild/moderate symptoms also showed elevated titers of IgG Ab. Therefore, it is not clear whether Ab titers correlate with the clinical progression of the patients. Several reports describe a positive correlation between clinical severity and Ab titers (*24*, *25*), whereas a contradictory report suggests that early seroconversion along with high Ab titers correlates with a less severe clinical course (*26*). In addition, there was a report claiming no association between comorbidity and Ab titer (*27*).

SARS-CoV-2 can cause a severe respiratory infection, which suggests that patients will need to produce both systemic and mucosal nAbs, including those of the IgA isotype, for protective immunity. Our results showed that *IGHV3-53/IGHV3-66* and *IGHJ6* class-switched to IgA1 in patients G, I, and J and class-switched to IgA1 and IgA2 in patient E (table S3). After 99 days from the onset of symptoms, we did not detect any RBD-reactive IGH clonotypes in the peripheral blood of patient E; however, the Ab titer to RBD remained high (Figs. 1 and 3D). This observation is in line with the findings that nAb titers remain detectable among a fraction of patients with SARS and MERS 1 to 2 years after infection (*28*, *29*). Therefore, it can be inferred that nAb-producing plasmablasts may be mobilized from the peripheral blood to bone marrow niches and continue to produce nAbs in patient E. In these niches, plasmablasts are able to differentiate into mature Ab-secreting plasma cells and can survive for decades (*30*).

In the rhesus macaque model of SARS-CoV-2 infection, viral rechallenge generated a greater nAb titer than induced by primary infection and protected animals from reinfection (*31*). A single case of SARS-CoV-2 reinfection that has been reported suggests that it is possible for humans to become infected multiple times by SARS-CoV-2 (*32*). It is still unclear whether a SARS-CoV-2 vaccine would provide prolonged protective immunity to humans. The long-term production of nAbs, as seen in patient E out to day 99 after symptom onset, suggests that nAb production by plasma cells in bone marrow niches may be a critical mechanism triggered by infection or vaccination that leads to protective immunity against SARS-CoV-2.

It has been reported that the frequency of RBD-reactive B cell clones is extremely low (0.07 to 0.005%) among circulating B cells (*24*). In our study, the frequency of isolated nAb clonotypes in the IGH repertoire was also extremely low (0.0004 to 0.0064%), and patient A had only one of six nAbs that mapped to the IGH repertoire (Fig. 2B). As the complexity of scFv phage display libraries exceeded 3.8 × 10^8^ and 6.7 × 10^8^ colony-forming units for patient A, diverse RBD-binding clones could be enriched by biopanning. Only 199,561 unique IGH sequences were sampled by NGS in patient A, whereas 515,994 IGH sequences were sampled in patient E, when scFv phage display libraries were constructed. This difference in NGS throughput might explain the discrepant allocation of nAb clonotypes in the IGH repertoire of patients A and E. Consistent with this hypothesis, only 38.3 and 22.0% of “bind” and “predicted” clones were mapped for patient A, respectively, whereas 77.8 and 32.1% of bind and predicted clones were individually mapped for patient E.

A major limitation of this study was the limited number of patients enrolled made it difficult to analyze whether these stereotypic nAb clonotypes affect the clinical course of COVID-19. We found that stereotypic nAb clonotypes preexisted in the majority of the naïve population were prevalent among the patients who displayed rapid class switching to IgG and IgA isotypes and exhibited light chain plasticity among the SARS-CoV-2 RBD-binding Abs. These results suggest that stereotypic nAb clonotypes may contribute to a milder clinical course and lower mortality rate in patients with SARS-CoV-2 compared with patients with SARS-CoV (9.5%) or MERS-CoV (34.4%) (*33*), for which similar stereotypic nAb clonotypes have not yet been reported. Future chronological follow-ups of Ig repertoires in a larger population of patients with SARS-CoV-2 may provide insight into the effects of stereotypic nAb clonotypes on patient outcomes. Furthermore, studies assessing changes in the Ig repertoire among the naïve populations after SARS-CoV-2 vaccination may provide details about the contribution of these stereotypic nAb clonotypes to protective immunity.

## MATERIALS AND METHODS

### Study design

This study was designed to investigate stereotypic nAb clonotypes of SARS-CoV-2. Twenty-six blood samples were collected from 17 SARS-CoV-2–positive patients and were subjected to NGS analysis of Ig sequences. Human Ab libraries were prepared and subjected to biopanning against recombinant SARS-CoV-2 RBD proteins. RBD binders were selected using ELISA, and their neutralization activity was tested using flow cytometry with ACE2-expressing Vero cells and recombinant SARS-CoV-2 S protein and microneutralization assay. NGS analysis of the enrichment patterns of clones through biopanning was performed for in silico selection of RBD-binding clones. Ig repertoire analyses were conducted to identify and characterize nAb clonotypes, including their prevalence among patients, frequency in Ig repertoires, somatic mutations, isotypes, chronological changes, and existence in a naïve uninfected population.

### Clinical protocol and blood sample collection

All patients were confirmed to be infected by SARS-CoV-2 by a positive reverse transcription quantitative polymerase chain reaction (RT-qPCR) result, and sample collection was performed at Seoul National University Hospital. The study involving human sample collection was approved by the Institutional Ethics Review Board of Seoul National University Hospital (IRB approval number, 2004-230-1119). Three chronological peripheral blood samples were drawn from patients A and E, and two chronological samples were obtained from patients B, C, D, F, and G. A single blood sample was collected from patients H to Q. Peripheral blood mononuclear cells (PBMCs) and plasma were isolated using Lymphoprep (STEMCELL Technologies, Vancouver, BC, Canada), according to the manufacturer’s protocol. PBMCs were subjected to total RNA isolation, using the TRI Reagent (Invitrogen, Carlsbad, CA, USA), according to the manufacturer’s protocol.

### Next-generation sequencing

Genes encoding V_H_ and part of the first constant domain of the heavy chain (CH1) domain were amplified, using specific primers, as described previously (*16*, *34*). All primers used are listed in table S8. Briefly, total RNA was used as a template to synthesize complementary DNA (cDNA), using the SuperScript IV First-Strand Synthesis System (Invitrogen), with specific primers targeting the constant region (CH1 domain) of each isotype (IgM, IgD, IgG, IgA, and IgE) (*34*), according to the manufacturer’s protocol. After cDNA synthesis, 1.8 volumes of SPRI beads (AMPure XP, Beckman Coulter, Brea, CA, USA) were used to purify cDNA, which was eluted in 40 μl of water. The purified cDNA (18 μl) was subjected to second-strand synthesis in a 25-μl reaction volume, using V gene–specific primers (*16*) and KAPA Biosystems (KAPA HiFi HotStart, Roche, Basel, Switzerland). The PCR conditions were as follows: 95°C for 3 min, 98°C for 1 min, 55°C for 1 min, and 72°C for 5 min. After the second-strand synthesis, double-stranded DNA (dsDNA) was purified, using SPRI beads, as described above. V_H_ genes were amplified using 15 μl of eluted dsDNA and 2.5 pmol of the primers listed in table S8, in a 50-μl total reaction volume (KAPA Biosystems), using the following thermal cycling program: 95°C for 3 min; 17 cycles of 98°C for 30 s, 65°C for 30 s, and 72°C for 1 min 10 s; and 72°C for 5 min. The number of PCR cycles was increased, from 17 to 19, for samples from patients B (d10 and d19), C (d6), E (d23), and G (d9 and d22). PCR products were purified using SPRI beads and eluted in 30 μl of water. Genes encoding V_κ_ and V_λ_ were amplified using specific primers, as described previously (*20*, *35*). Briefly, total RNA was used as a template to synthesize cDNA, using the SuperScript IV First-Strand Synthesis System (Invitrogen), with specific primers targeting the constant region, which are listed in table S8, according to the manufacturer’s protocol. After cDNA synthesis, SPRI beads were used to purify cDNA, which was eluted in 40 μl of water. Purified cDNA (18 μl) was used for the first amplification, in a 25-μl reaction volume, using VJ gene–specific primers, which are listed in table S8, and KAPA Biosystems. The PCR conditions were as follows: 95°C for 3 min; four cycles of 98°C for 1 min, 55°C for 1 min, and 72°C for 1 min; and 72°C for 10 min. Subsequently, DNA was purified using SPRI beads, and the V_κ_ and V_λ_ genes were amplified using 15 μl of eluted dsDNA and 2.5 pmol of the primers listed in table S8, in a 50-μl total reaction volume (KAPA Biosystems). The PCR conditions were as follows: 95°C for 3 min; 17 cycles of 98°C for 30 s, 65°C for 30 s, and 72°C for 1 min 10 s; and 72°C for 5 min. PCR products were purified using SPRI beads, as described above. For the amplification of V_H_ from each round of biopanning (rounds 0 to 4), gene fragments were amplified from phagemid DNA, using the primers listed in table S8. SPRI-purified sequencing libraries were quantified with a 4200 TapeStation System (Agilent Technologies), using a D1000 ScreenTape assay, before performing sequencing on an Illumina MiSeq platform.

### NGS data processing

#### Preprocessing of the NGS data for the Ig repertoire

The raw NGS forward (R1) and reverse (R2) reads were merged by paired-end read merger (PEAR), v0.9.10, in default setting (*36*). The merged reads were q-filtered using the condition q20p95, which results in 95% of the base pairs in a read having Phred scores higher than 20. The location of the primers was recognized from the q-filtered reads while allowing one substitution or deletion (table S8). Primer regions that specifically bind to the molecules were trimmed in the reads to eliminate the effects of primer synthesis errors. On the basis of the primer recognition results, unique molecular identifier (UMI) sequences were extracted, and the reads were clustered according to the UMI sequences. To eliminate the possibility that the same UMI sequences might be used for different read amplifications, the clustered reads were subclustered, according to the similarity of the reads (five mismatches were allowed in each subcluster). The subclustered reads were aligned using a multiple sequence alignment tool, Clustal Omega, v1.2.4, in default setting (*37*, *38*). From the aligned reads, the frequency of each nucleotide was calculated, and a consensus sequence of each subcluster was defined using the frequency information. The read count of the consensus sequence was redefined as the number of UMI subclusters that belong to the consensus sequences.

#### Sequence annotation, functionality filtering, and throughput adjustment

Sequence annotation consisted of two parts: isotype annotation and V(D)J annotation. For annotation, the consensus sequence was divided into two sections, a V(D)J region and a constant region, in a location-based manner. For isotype annotation, the extracted constant region was aligned with the IMGT (International Immunogenetics Information System) constant gene database (*39*). On the basis of the alignment results, the isotypes of the consensus sequences were annotated. Then, the V(D)J regions of the consensus sequences were annotated using IgBLAST, v1.8.0 (*40*). Among the annotation results, V(D)J genes, CDR1/2/3 sequences, and the number of mutations from the corresponding V genes were extracted for further analysis. Divergence values were defined as the number of mutations identified in the aligned V gene, divided by the aligned length. The nonfunctional consensus reads were defined using the following criteria and filtered out: (i) sequence length shorter than 250 base pairs, (ii) existence of stop codon or frame shift in the full amino acid sequence, (iii) annotation failure in one or more of the CDR1/2/3 regions, and (iv) isotype annotation failure. The functional consensus reads were randomly sampled to adjust the throughput of the V_H_ data (table S5). Throughput adjustment was not conducted for V_λ_ data (table S6).

#### Preprocessing of the biopanning NGS data

Preprocessing of the biopanning NGS data was performed as previously reported, except for the application of the q-filtering condition q20p95 instead of q20p100 (*41*).

#### Overlapping IGH repertoire construction

To investigate the shared IGH sequences among the patients, we defined the overlapping IGH repertoire of the patients. Histograms for the nearest-neighbor distances of the HCDR3 amino acid sequences were calculated for the repertoire data. A hierarchical, distance-based analysis, which was reported previously (*42*), was applied to the HCDR3 amino acid sequences to cluster functionally similar IGH sequences. The IGH sequences for all repertoire data could be approximated into a bimodal distribution, allowing the functionally similar IGH sequences to be extracted by capturing the first peak of the distribution (fig. S11). Threshold values for each dataset were defined as the nearest-neighbor distance value of those points with a minimum frequency between the two peaks of the distribution. Then, the minimum value among all threshold values, 0.113871, was used to construct the overlapping IGH repertoire, which means that 11.3871% of mismatches in the HCDR3 amino acid sequence were allowed in the overlapping IGH repertoire construction. To construct the overlapping IGH repertoire, the repertoire datasets of all patients were merged into one dataset. The IGH sequences in the merged dataset were then clustered using the following conditions: (i) the same V and J gene usage and (ii) mismatch smaller than 11.3871% among the HCDR3 amino acid sequences. Subsequently, clusters containing IGH sequences from more than one patient were included in the overlapping IGH repertoire dataset.

#### Extraction of binding-predicted clones

From each round of biopanning (rounds 0, 2, 3, and 4), the V_H_ genes were amplified and subjected to NGS analysis, using the MiSeq platform, as described previously (*19*). Binding-predicted clones from biopanning were selected by analyzing the enrichment or diminishment patterns of clones in the NGS data from four libraries, A_d17, A_d45, E_d23, and E_d44, at each round of biopanning. The enrichment of clones primarily occurred during the second round of biopanning based on the input/output virus titer values for each round of biopanning and the frequencies of the clones in the NGS data (fig. S12). The frequency information in the NGS datasets for biopanning rounds 0, 2, 3, and 4 was subject to principal components analysis (PCA) for dimension reduction. Accordingly, principal component 1 (PC1) and PC2, which represented clone enrichment and clone depletion, respectively, were extracted. In the biopanning data, PC1 was primarily composed of the frequencies in rounds 2, 3, and 4, whereas PC2 was primarily composed of the frequency in round 0 (fig. S13). Thus, we defined PC1 major clones as the predicted clones by setting constant threshold values on the PC1 value and the ratio between PC1 and PC2 (table S7). RBD-binding clones (94.74%) were mapped to the predicted clones (fig. S13).

#### Construction of a human scFv phage display library and V_λ_ shuffled libraries

For the V_H_ gene, the cDNA prepared for the NGS analysis was used. For the V_κ_ and V_λ_ genes, total RNA was used to synthesize cDNA, using the SuperScript IV First-Strand Synthesis System (Invitrogen), with oligo(dT) primers, according to the manufacturer’s instructions. Then, the genes encoding V_κ_/V_λ_ and V_H_ were amplified, from the oligo(dT)-synthesized cDNA and the cDNA prepared for NGS analysis, respectively, using the primers listed in table S8 and KAPA Biosystems. The PCR conditions were as follows: preliminary denaturation at 95°C for 3 min; four cycles of 98°C for 1 min, 55°C for 1 min, and 72°C for 1 min; and 72°C for 10 min. Subsequently, DNA was purified using SPRI beads, as described above. The purified DNA was amplified using the primers listed in table S8 and KAPA Biosystems. The PCR conditions were as follows: preliminary denaturation at 95°C for 3 min; 25 cycles of 98°C for 30 s, 58°C for 30 s, and 72°C for 90 s; and 72°C for 10 min. Then, the V_H_ and V_κ_/V_λ_ fragments were subjected to electrophoresis, on a 1% agarose gel, and purified, using a QIAquick gel extraction kit (QIAGEN Inc., Valencia, CA, USA), according to the manufacturer’s instructions. The purified V_H_ and V_κ_/V_λ_ fragments were mixed, at equal ratios at 50 ng, and subjected to overlap extension to generate scFv genes, using the primers listed in table S8 and KAPA Biosystems. The PCR conditions were as follows: preliminary denaturation at 94°C for 5 min; 25 cycles of 98°C for 15 s, 56°C for 15 s, and 72°C for 2 min; and 72°C for 10 min. The amplified scFv fragment was purified and cloned into a phagemid vector, as described previously (*43*).

For the construction of V_κ_/V_λ_ shuffled libraries, gBlocks Gene Fragments (Integrated DNA Technologies, Coralville, IA, USA), encoding A-11, E-12, A-31, A-32, B-33, E-34, A,B,G-42, G-44, D-51, F-53, E-52, and A-54, were synthesized. Synthesized V_H_ and the V_κ_/V_λ_ genes from patients A, E, and G were used to synthesize the scFv libraries using PCR, as described previously (*43*). Then, the amplified scFv fragments were purified and cloned into the phagemid vector, as described above.

#### Biopanning

Phage display of the human scFv libraries exceeded complexity of 3.8 × 10^8^, 6.7 × 10^8^, 2.0 × 10^8^, and 7.2 × 10^8^ colony-forming units for A_d17, A_d45, E_d23, and E_d44, respectively. These libraries were subjected to four rounds of biopanning against the recombinant SARS-CoV-2 RBD protein (Sino Biological Inc., Beijing, China), fused to mouse Fc or hCκ, as described previously (*44*). Briefly, 3 μg of the recombinant SARS-CoV-2 RBD protein was conjugated to 1.0 × 10^7^ magnetic beads (Dynabeads M-270 epoxy, Invitrogen) and incubated with the scFv phage display libraries (about 10^12^ phages), for 2 hours at 37°C. During the first round of biopanning, the beads were washed once with 500 μl of 0.05% (v/v) Tween 20 (Sigma-Aldrich, St. Louis, MO, USA) in phosphate-buffered saline (PBST). For the other rounds of biopanning, 1.5 μg of recombinant SARS-CoV-2 RBD protein was conjugated to 5.0 × 10^6^ magnetic beads, and the number of washes was increased to three. After each round of biopanning, the bound phages were eluted and rescued, as described previously (*44*).

#### Phage ELISA

To select SARS-CoV-2 S reactive clones, phage ELISA was performed, using recombinant S and RBD protein–coated microtiter plates, as described previously (*45*). Reactive scFv clones were subjected to Sanger sequencing (Cosmogenetech, Seoul, Republic of Korea) to determine their nucleotide sequences.

#### Expression of recombinant proteins

A human, codon-optimized, SARS-CoV-2 RBD (YP_009724390.1, amino acids 306 to 543) gene was synthesized (Integrated DNA Technologies). Using a synthesized wild-type RBD gene as a template, RBD mutants (V341I, F342L, N354D, N354D/D364Y, V367F, R408I, A435S, W436R, G476S, and V483A) were generated through two-step PCR, using the primers listed in table S8. The genes encoding wild-type or mutant SARS-CoV-2 RBD were cloned into a modified mammalian expression vector, containing the hCκ gene (*44*), and transfected into Expi293F (Invitrogen) cells. The fusion proteins were purified by affinity chromatography, using KappaSelect columns (GE Healthcare, Chicago, IL, USA), as described previously (*46*). Because of low expression yields, two RBD mutants (N354D/D364Y and W436R) were excluded from further studies.

The genes encoding the selected scFv clones were cloned into a modified mammalian expression vector, containing the hFc or hCκ at the C terminus (*44*, *47*), before being transfected and purified by affinity chromatography, as described above. Genes encoding V_H_ and V_λ_ were amplified, cloned into a mammalian expression vector containing the CH1 and hinge regions of human IgG2 fused to the CH2 and CH3 regions of human IgG4 (*48*, *49*), and transfected into Expi293F cells (Invitrogen) as described previously (*50*). Then, IgG2/4 was purified by affinity chromatography using MabSelect columns with the AKTA pure chromatography system (GE Healthcare) following the manufacturer’s protocol.

### Enzyme-linked immunosorbent assay

One hundred nanograms of each recombinant SARS-CoV-2 S (Sino Biological Inc.), S1 (Sino Biological Inc.), S1 D614G (Sino Biological Inc.), S2 (Sino Biological Inc.), nucleocapsid (N) (Sino Biological Inc.), RBD, RBD mutants, SARS-CoV RBD (Sino Biological Inc.), MERS-CoV S (Sino Biological Inc.), RBD (Sino Biological Inc.), and S2 (Sino Biological Inc.) proteins were added to microtiter plates (CoStar), in coating buffer (0.1 M sodium bicarbonate, pH 8.6). After incubation at 4°C overnight and blocking with 3% bovine serum albumin (BSA) in PBS, for 1 hour at 37°C, serially diluted plasma (fivefold, six dilutions, starting from 1:100) or scFv-hFc (fivefold, 12 dilutions, starting from 1000 or 500 nM) in blocking buffer was added to individual wells and incubated for 1 hour at 37°C. Then, plates were washed three times with 0.05% PBST. Horseradish peroxidase (HRP)–conjugated rabbit anti-human IgG Ab (Invitrogen) or anti-human Ig κ light chain Ab (Millipore, Temecula, CA, USA), in blocking buffer (1:5000), was added to wells and incubated for 1 hour at 37°C. After washing three times with PBST, 2,2′-azino-bis-3-ethylbenzothiazoline-6-sulfonic (Thermo Fisher Scientific Inc., Waltham, MA, USA) or 3,3′,5,5′-tetramethylbenzidine liquid substrate system (Thermo Fisher Scientific Inc.) was added to the wells. Absorbance was measured at 405 or 650 nm, respectively, using a microplate spectrophotometer (Multiskan GO, Thermo Fisher Scientific Inc.).

### Flow cytometry

The recombinant SARS-CoV-2 S protein (200 nM), fused with a HIS tag at the C terminus (Sino Biological Inc.), was incubated with scFv-hFc fusion proteins at a final concentration of either 200 nM (equimolar) or 600 nM (molar ratio of 1:3), in 50 μl of 1% (w/v) BSA in PBS, containing 0.02% (w/v) sodium azide [flow cytometry staining buffer (FCSB)], at 37°C for 1 hour. Irrelevant scFv-hFc or scFv-hCκ fusion proteins were used as negative controls. Vero E6 cells (ACE2^+^) were seeded into v-bottom 96-well plates (Corning, Corning, NY, USA) at a density of 1.5 × 10^5^ cells per well. Then, the mixture was added to each well and incubated at 37°C for 1 hour. After washing three times with FCSB, FITC-labeled rabbit anti-HIS Ab (Abcam, Cambridge, UK) was incubated at 37°C for 1 hour. Then, the cells were washed three times with FCSB, resuspended in 150 μl of PBS, and subjected to analysis by flow cytometry, using a FACSCanto II instrument (BD Bioscience, San Jose, CA, USA). For each sample, 10,000 cells were analyzed.

### Microneutralization assay

The virus (BetaCoV/Korea/SNU01/2020, accession number MT039890) was isolated at Seoul National University Hospital and propagated in Vero cells (American Type Culture Collection CCL-81) using Dulbecco’s modified Eagle’s medium (DMEM; WELGENE, Gyeongsan, Republic of Korea) supplemented with 2% fetal bovine serum (Gibco) (*51*). The cells were grown in T-25 flasks (Thermo Fisher Scientific Inc.), inoculated with SARS-CoV-2, and incubated at 37°C in a 5% CO_2_ environment. Three days after inoculation, virus was harvested from culture supernatants and stored at −80°C. The virus titer was determined via a TCID_50_ assay (*52*).

Vero cells were seeded in T-25 flasks and grown for 24 hours at 37°C in a 5% CO_2_ environment to ensure 80% confluency on the day of inoculation. The recombinant scFv-hCκ fusion proteins (0.5, 5, or 50 μg/ml) were mixed with 2500 TCID_50_ of SARS-CoV-2, and the mixture was incubated for 2 hours at 37°C. The mixture (1 ml) was added to Vero cells and incubated for 1 hour at 37°C in a 5% CO_2_ environment. After incubation for 1 hour, 6 ml of complete media were added to the flasks and incubated at 37°C in a 5% CO_2_ environment. After 0, 24, 48, and 72 hours of infection, the culture supernatant was collected to measure the virus titers. Viral RNA was extracted using the MagNA Pure 96 DNA and Viral NA small volume kit (Roche, Germany), according to the manufacturer’s instructions. Viral RNA was detected using the PowerChek 2019-nCoV Real-Time PCR Kit (Kogene Biotech, Seoul, Republic of Korea) for the amplification of the envelope (E) gene and quantified according to a standard curve, which was constructed using in vitro transcribed RNA provided by the European Virus Archive (www.european-virus-archive.com). An alternative neutralization assay was performed as described previously (*10*). Briefly, Vero cells that were seeded in 96-well flat-bottom tissue culture microtiter plates in DMEM were grown for 24 hours at 37°C in a 5% CO_2_ environment. Fifty microliters of twofold serially diluted IgG2/4 were mixed with an equal volume of SARS-CoV-2 containing 100 TCID_50_, and the IgG2/4-virus mixture was incubated at 37°C for 1 hour. The mixture was then transferred into a 96-well microtiter plate containing Vero cells with eight repeats and incubated for 5 days at 37°C in a 5% CO_2_ environment. Cells infected with 100 TCID_50_ of SARS-CoV-2, isotype IgG2/4 control, or without the virus were applied as positive, negative, and uninfected controls, respectively. The cytopathic effect (CPE) in each well was observed 5 days after infection. The IC_50_ was calculated using GraphPad Prism 8 (GraphPad Software, San Diego, CA, USA). All experiments using authentic SARS-CoV-2 were conducted in biosafety level 3 laboratory.

### Statistical analyses

Data are represented as means ± SD. Statistical analyses were performed using R software version 3.4.3. For the flow cytometry analysis using ACE2-expressing cells and recombinant SARS-CoV-2 S protein, results were analyzed by independent *t* tests.

## SUPPLEMENTARY MATERIALS

stm.sciencemag.org/cgi/content/full/scitranslmed.abd6990/DC1 Fig. S1. Titrations of serum IgG by ELISAs specific to MERS-CoV. Fig. S2. Reactivity of anti–SARS-CoV-2 scFv Abs against recombinant SARS-CoV-2 RBD. Fig. S3. Inhibition of recombinant SARS-CoV-2 S glycoprotein binding to ACE2-expressing cells. Fig. S4. In vitro neutralization of SARS-COV-2. Fig. S5. Mapping of 11 nAbs to the overlapping IGH repertoire. Fig. S6. Existence of V_λ_ that can be paired with the stereotypic V_H_. Fig. S7. VJ gene usage among the Igκ light chain repertoire of patients. Fig. S8. VJ gene usage among the Igλ light chain repertoire of patients. Fig. S9. Reactivity of phage-displayed scFv clones in phage ELISA. Fig. S10. Deep profiling of the IGH repertoire of patients B, C, D, F, and G. Fig. S11. The nearest-neighbor distance histogram for HCDR3 amino acid sequences in the IGH repertoires of patients. Fig. S12. Frequency scatter plots for NGS data from four libraries after each round of biopanning. Fig. S13. The results of PCA applied to the NGS data of four libraries after each round of biopanning. Table S1. Demographic and clinical characteristics. Table S2. SARS-CoV-2 RBD-reactive scFv clones. Table S3. Class-switched IGH clonotypes homologous to E-3B1. Table S4. Human mAbs reactive against MERS-CoV RBD. Table S5. Statistics for the preprocessing of the IGH NGS data. Table S6. Statistics for the preprocessing of the Igκ and Igλ NGS data. Table S7. The RBD-binding prediction clones. Table S8. Primers used in the study. Data file S1.

## Appendix

View/request a protocol for this paper from *Bio-protocol*.
